# A Novel Audiovisual Brain-Computer Interface and Its Application in Awareness Detection

**DOI:** 10.1038/srep09962

**Published:** 2015-06-30

**Authors:** Fei Wang, Yanbin He, Jiahui Pan, Qiuyou Xie, Ronghao Yu, Rui Zhang, Yuanqing Li

**Affiliations:** 1 Center for Brain Computer Interfaces and Brain Information Processing, South China University of Technology, Guangzhou 510640, China; 2 Coma Research Group, Centre for Hyperbaric Oxygen and Neurorehabilitation, Guangzhou General Hospital of Guangzhou Miliatry Command, Guangzhou 510010, China; 3 Guangzhou Key Laboratory of Brain Computer Interaction and Applications, Guangzhou 510640, China

## Abstract

Currently, detecting awareness in patients with disorders of consciousness (DOC) is a challenging task, which is commonly addressed through behavioral observation scales such as the JFK Coma Recovery Scale-Revised. Brain-computer interfaces (BCIs) provide an alternative approach to detect awareness in patients with DOC. However, these patients have a much lower capability of using BCIs compared to healthy individuals. This study proposed a novel BCI using temporally, spatially, and semantically congruent audiovisual stimuli involving numbers (i.e., visual and spoken numbers). Subjects were instructed to selectively attend to the target stimuli cued by instruction. Ten healthy subjects first participated in the experiment to evaluate the system. The results indicated that the audiovisual BCI system outperformed auditory-only and visual-only systems. Through event-related potential analysis, we observed audiovisual integration effects for target stimuli, which enhanced the discriminability between brain responses for target and nontarget stimuli and thus improved the performance of the audiovisual BCI. This system was then applied to detect the awareness of seven DOC patients, five of whom exhibited command following as well as number recognition. Thus, this audiovisual BCI system may be used as a supportive bedside tool for awareness detection in patients with DOC.

Brain-computer interfaces (BCIs) provide a direct method of communication between the brain and an external device^1^,^2^. The features of scalp recorded EEG signals, commonly used in BCIs, include P300 potentials^3^, steady state visual evoked potentials (SSVEPs)^4^, N200^5^,^6^, and mu/beta rhythms^7^. P300 potentials can be elicited by an oddball paradigm. Specifically, the subject is required to focus on visual/auditory target stimuli that are hidden as rare occurrences among a series of more common nontarget stimuli. In this case, an event-related potential (ERP), i.e., P300, across the parieto-central area of the skull may occur about 300 ms after the target stimulus^8^. P300, by which the target stimuli can be identified, has been commonly used in visual/auditory/audiovisual BCIs.

For normal subjects, many visual P300 BCIs have been developed to provide efficient control of external devices. For instance, several visual BCI spellers based on P300 have been reported^9^,^10^. Li *et al.* proposed a visual hybrid BCI combining P300 and SSVEP^11^. Regarding auditory BCIs, Furdea *et al.* proposed a spelling system in which the letters in a 5 × 5 matrix were coded with acoustically presented numbers^12^. Schreuder *et al.* proposed an auditory spatial ERP paradigm involving spatially distributed auditory cues^13^. These BCIs are typically based on auditory ERPs, including P300. However, existing results suggest that auditory-only BCI systems generally perform worse than visual BCIs. Several audiovisual BCIs have been reported in recent years. Sellers *et al.* evaluated the effectiveness of a P300-based BCI that could use four different types of visual, auditory, or audiovisual stimuli (i.e., YES, NO, PASS and END)^14^. Offline analysis results showed that the auditory mode did not reach the same level of classification accuracy as the visual or audiovisual mode did and that the performance of the audiovisual mode was not significantly better than that of the visual mode. Belitski *et al.* proposed an offline audiovisual P300-speller and found that audiovisual stimulation increased the average strength of the response compared to that of either visual-only or auditory-only stimulation^15^.

When receiving spatially, temporally, and semantically congruent audiovisual stimuli, our brains may integrate the auditory and visual features of these stimuli^16^,^17^. Single-cell studies^18^,^19^ and functional magnetic resonance imaging (fMRI) experiments^20^,^21^,^22^ have demonstrated that neural responses in heteromodal brain areas, including the posterior superior temporal sulcus/middle temporal gyrus (pSTS/MTG), are more pronounced for congruent audiovisual stimuli than for corresponding visual-only and auditory-only stimuli. In previous EEG studies^23^,^24^,^25^,^26^,^27^,^28^, audiovisual integration processes were assessed by comparing the ERPs elicited by audiovisual stimuli with the sum of the ERPs elicited by visual-only and auditory-only stimuli (i.e., AV vs. A + V). Teder *et al.* reported both early (at approximately 40 ms for sites Fz, Pz) and late (after 100 ms for sites Fz, Cz and Pz) ERP enhancement effects of audiovisual integration^29^. Talsma *et al.* observed multisensory integration effects at approximately 100 ms (frontal positivity), 160 ms (centro-medial positivity), 250 ms (centro-medial negativity), and 300–500 ms (centro-medial positivity)^30^. Based on the observations reported in the study^30^, we conjecture that the P300 could be enhanced by combing the paradigm eliciting P300 and that for audiovisual integration. Furthermore, these enhanced ERPs in the audiovisual condition may be used to develop novel audiovisual BCIs that have not been sufficiently considered in existing audiovisual BCI studies^14^,^15^.

A BCI system can detect command/intention-specific changes in EEG signals and thus allow patients who have lost movement ability after an injury or disease to convey their intent to the external world^1^,^2^. One potential application of BCIs is awareness detection in patients with disorders of consciousness (DOC), such as vegetative state (VS; also called unresponsive wakefulness syndrome (UWS)), minimally conscious state (MCS), emerged from MCS (EMCS) and locked-in syndrome (LIS). Some patients with DOC may be diagnosed with VS, in which they may awaken but show no awareness of themselves or their environment^31^. Other patients may improve to MCS, in which they demonstrate inconsistent but reproducible signs of awareness^32^. Furthermore, EMCS is characterized by reliable and consistent demonstration of functional interactive communication or functional use of two different objects^33^. Recently, Bruno *et al*^34^. proposed to subdivide MCS patients into patients responding to commands (MCS+) and patients showing only non-reflex behavior (MCS−). Currently, the clinical diagnosis of patients with DOC is generally based on behavioral observation scales, including the Glasgow Coma Scale (GCS) and the JFK Coma Recovery Scale-Revised (JFK CRS-Revised). Because these patients cannot provide sufficient behavioral responses, misdiagnosis rates in VS and MCS patients range from 37 to 43%^35^,^36^,^37^. Several BCI paradigms have been presented for patients with DOC. Lule *et al.* tested a four-choice auditory oddball BCI on 16 healthy subjects and 18 patients with DOC (13 MCS, 3 VS and 2 LIS)^38^. Their results showed that 13 healthy subjects, 1 MCS patient and 1 LIS patient were able to communicate using the BCI. Coyle *et al.* reported an MCS patient who could use a motor imagery (MI)-based BCI system with 80% online accuracy^39^. Pan *et al.* developed a visual hybrid BCI combining P300 and SSVEP to detect awareness in eight patients with DOC (4 VS, 3 MCS, and 1 LIS) and successfully demonstrated command following in three patients (1 VS, 1 MCS, and 1 LIS)^40^. Considering that training for MI-based BCI is a heavy burden, P300-based BCIs may be more suitable for patients with DOC.

However, BCI-based awareness detection in patients with DOC is still in its infancy. The performance of the BCIs designed for these patients is generally poor because the patients’ cognitive ability is considerably lower than that of healthy individuals. Furthermore, most of these patients lack control of gaze movement. One possible solution is to develop novel BCIs to improve sensitivity in awareness detection. In this study, we proposed a novel audiovisual BCI system in which spatially, temporally and semantically congruent audiovisual stimuli of numbers were employed. The system was first tested by both online and offline analyses in ten healthy subjects and compared to both visual-only and auditory-only systems. We then applied our audiovisual BCI system for online awareness detection in seven patients with DOC.

## Results

### Classification results for healthy subjects

**Figure 1:**
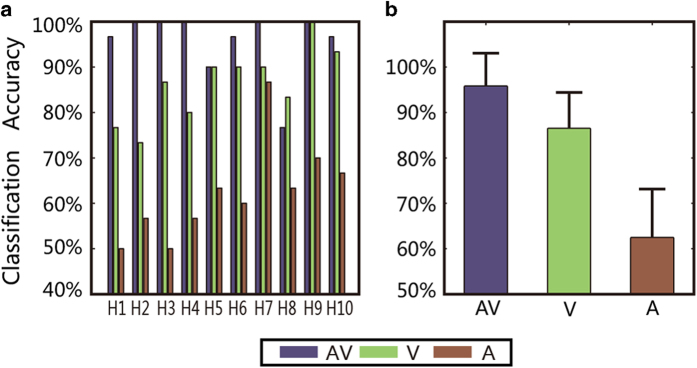
Online classification accuracies of the three sessions (audiovisual, visual-only and auditory-only) for healthy subjects. (**a**) Accuracies of three sessions for each healthy subject. (**b**) Average accuracy with the standard deviation value in each session.

Figure 1(a) summarizes the online classification accuracies obtained for the healthy subjects. For each healthy subject, the auditory-only experiment exhibited the lowest accuracy. The audiovisual accuracies for nine of the ten healthy subjects were better than or equal to the visual-only accuracies. The average accuracy across all subjects in each stimulus condition is presented in Fig. 1(b); as shown, the average accuracy rates in the audiovisual (AV), visual-only (V) and auditory-only (A) conditions were 95.67%, 86.33%, and 62.33% respectively. For the healthy subjects, the audiovisual BCI exhibited the best performance according to our t-test (two-tailed) (audiovisual vs. auditory-only: t[9] = 8.076, *p* < 0.0001; audiovisual vs. visual-only: t[9] = 2.775, *p* < 0.03).

**Figure 2:**
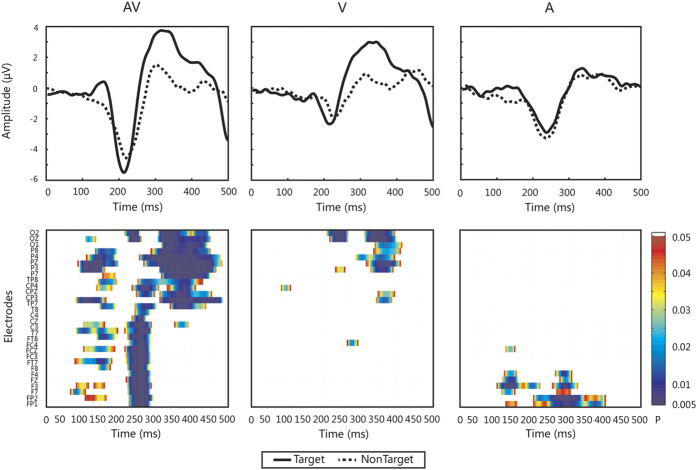
ERP waveforms and comparison results. Upper panel: Average ERP waveforms of all healthy subjects in each stimulus condition from the “Pz” electrode. The solid and dashed curves correspond to the target and nontarget stimuli, respectively. Lower panel: Point-wise running t-tests compared target with nontarget responses in multisensory and unisensory stimulus conditions across all healthy subjects for 30 electrodes. Significant differences were plotted when data points met an alpha criterion of 0.05 with a cluster size larger than 7.

We compared the responses for target and nontarget stimuli in the audiovisual, visual-only and auditory-only conditions in our ERP analysis. The group average ERP waveforms at “Pz” are shown in the upper panel of Fig. 2, and the statistical analysis based on t-test indicated the following: (i) for the target stimuli, stronger P100, N200 and P300 responses were recorded in the audiovisual condition than in the visual-only condition (P100: t[9] = 2.5361, *p* < 0.03; N200: t[9] = −3.263, *p* < 0.01; P300: t[9] = 2.1305, *p* < 0.05), or auditory-only condition (P100: t[9] = 2.2054, *p* < 0.03; N200: t[9] = −3.4653, *p* < 0.01; P300: t[9] = 5.0328, *p* < 0.001); (ii) for the nontarget stimuli, only the N200 response was stronger in the audiovisual condition than in the visual-only condition (N200: t[9] = −3.4872, *p* < 0.01); (iii) in the audiovisual condition, the P100 and P300 responses were stronger for the target stimuli than for the nontarget stimuli (P100: t[9] = 2.3982, *p* < 0.03; P300: t[9] = 5.5769, *p* < 0.001); (iv) in the visual-only condition, the P300 response was stronger for the target stimuli than for the nontarget stimuli (P300: t[9] = 3.7075, *p* < 0.01); and (v) in the auditory-only condition, both the target and nontarget stimuli elicited a P300 response, but the responses could not be distinguished at a significant level. We further evaluated the discriminative features using point-wise running t-tests (two-tailed) for target vs. nontarget responses in the three stimulus conditions. It follows from the lower panel of Fig. 2 that there were more discriminative features within certain time windows, such as 100–200, 200–300 and 300–500 ms, for the audiovisual condition than for the visual-only and auditory-only conditions.

Table 1 shows the classification accuracies obtained by using the features in three time windows corresponding to the three ERP components P100, N200 and P300. The results in Table 1 indicate the following: (i) in the audiovisual stimulus condition, the classification accuracy for each component was not less than 84%; (ii) the classification accuracy for each time window was higher for the audiovisual stimuli than for the auditory-only and visual-only stimuli; and (iii) over the entire time window (0–500 ms), the audiovisual stimuli exhibited the highest classification accuracy.

### Effects of audiovisual integration

**Figure 3:**
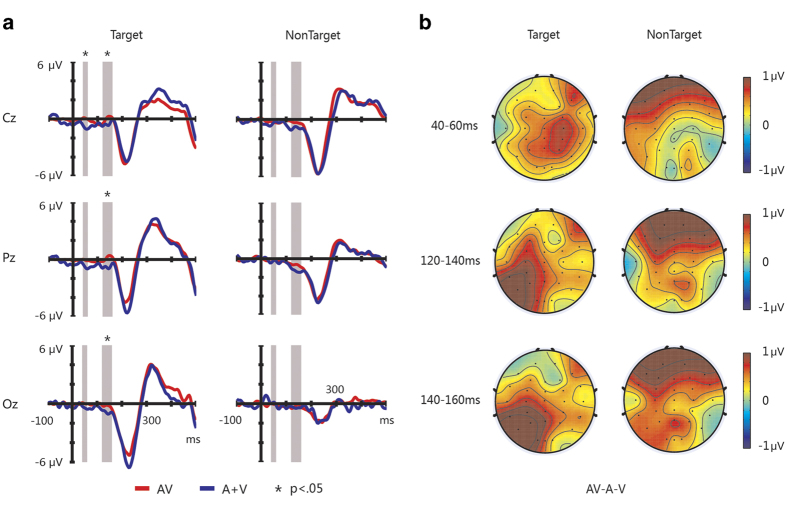
Effects of audiovisual integration for the target and nontarget stimuli. (**a**) ERPs for the target and nontarget stimuli. Audiovisual ERPs are shown in red, and A + V ERPs are shown in blue. The time windows of interest (i.e., 40–60, 120–140, and 140–160 ms) are shaded in gray. (**b**) Topographic maps of the difference ERPs (AV-A-V) for target and nontarget stimuli.

Inspired by previous studies^28^,^41^, we calculated the AV and A + V ERPs at three electrodes “Cz”, “Pz”, and “Oz”, which are illustrated in Fig. 3. Furthermore, we compared the AV and A + V signal amplitudes over successive 20 ms intervals from -100 to 500 ms after stimulus onset by applying t-tests (two-tailed) to the data of all healthy subjects. For target stimuli, the significant effects of audiovisual integration were observed over three time windows (i.e., the amplitudes were greater for AV responses than for A + V responses): 1) 40–60 ms (electrode “Cz”, t[9] = 2.551, *p* < 0.05); 2) 120–140 ms (electrode “Cz”, t[9] = 2.458, *p* < 0.05; electrode “Pz”, t[9] = 3.119, *p* < 0.03; and electrode “Oz”, t[9] = 2.449, *p* < 0.05); and 3) 140–160 ms (electrode “Cz”, t[9] = 2.565, *p* < 0.05; electrode “Pz”, t[9] = 3.524, *p* < 0.01; and electrode “Oz”, t[9] = 2.381, *p* < 0.05). We did not observe any significant effects of audiovisual integration for the nontarget stimuli (Fig. 3). The topographic maps of the difference ERPs (AV-A-V) for target and nontarget stimuli in the three windows are shown in Fig. 3.

### Patients’ results

**Figure 4:**
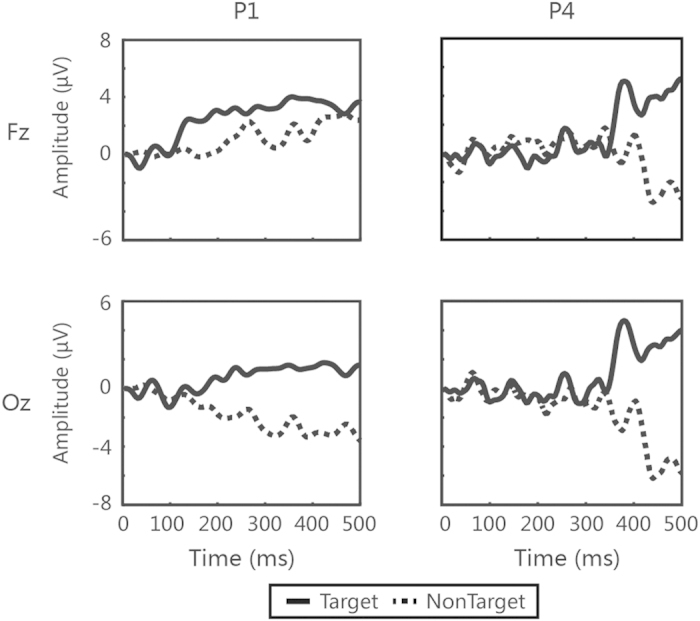
ERPs waveforms from the “Fz” and “Oz” electrodes for patients P1 and P4. The solid curves correspond to the target stimuli, and the dashed curves correspond to the nontarget stimuli.

The online results for the patients are shown in Table 2. Most of the patients performed at least 50 trials, except for patients P3 and P6 (who finished 40 trials and then left the hospital for financial reasons). Five of the seven patients (P1, P4, P5, P6 and P7) achieved accuracies (ranging from 66 to 74%) that were significantly higher than the chance level (*p* < 0.05, binomial test). The four MCS patients showing command following in our experiment could be diagnosed as MCS+, according to the subdivision criterion used in the study^34^. For example, for patients P1 and P4, who obtained accuracies of 66% and 74%, respectively, we calculated ERP waveforms from 0 to 500 ms after stimulus onset by averaging the EEG channel signal across all 50 trials. Fig. 4 shows the ERP waveforms measured at the electrodes “Fz” and “Oz”; this figure demonstrates a robust P300 response elicited by the target stimuli and highly similar non-P300 responses for the nontarget stimuli.

## Discussion

In this study, we proposed a novel audiovisual BCI system for awareness detection in patients with DOC based on semantically congruent audiovisual stimuli involving numbers. With respect to classification accuracy, the experimental results for healthy subjects showed that the audiovisual BCI system outperformed the visual-only and auditory-only BCI systems. The improved performance of the audiovisual BCI may be associated with audiovisual integration. Seven patients with DOC participated in the awareness detection experiment. To some extent, command following as well as number recognition were demonstrated in five patients, who obtained accuracies that were significantly higher than the chance level. Currently, the clinical misdiagnosis rates in VS and MCS patients range from 37 to 43%^35^,^36^,^37^. Such rates are observed because the clinical diagnosis of patients with DOC is generally based on behavioral observation scales such as GCS and JFK CRS-Revised and because these patients cannot provide sufficient behavioral responses. The proposed audiovisual BCI system, which shows comparatively high performance for awareness detection may provide a potential solution to this problem.

Our experimental results for healthy subjects show that audiovisual integration effects occurred for target stimuli. As shown in Fig. 3, the audiovisual interaction effects first included positivity with a latency of 40–60 ms at electrode “Cz” for target stimuli. Furthermore, a positive potential in the 120–160 ms interval was observed at electrodes “Cz”, “Pz” and “Oz”. These results are consistent with previous reports on audiovisual processing. Specifically, Teder-Salejarvi *et al.* observed an audiovisual interaction with a positivity that began at approximately 130 ms and peaked at 160–170 ms at electrodes “Fz”, “Cz” and “Pz”^29^. Talsma *et al.* observed audiovisual integration effects, including a P50 component, at electrodes “Fz”, “FCz” and “Cz”^28^, a frontal positivity at approximately 100 ms and a centro-medial positivity at approximately 160 ms^30^. Senkowski *et al.* observed an audiovisual interaction with positivity in short latency (40–60 ms) ERPs with left posterior and right anterior topographies^42^.

Furthermore, we observed audiovisual integration effects for the attended target stimuli but not for the nontarget stimuli (see Fig. 3). This observation is consistent with previous studies that demonstrated the effects of attention on audiovisual integration^23^,^28^,^30^. For instance, Talsma *et al.* found that audiovisual integration effects were larger in amplitude for attended than for unattended stimuli^30^. We also found that these multisensory integration effects influenced by attention not only enhanced the ERP components but also improved the performance of the BCI system. Specifically, as shown in Fig. 2, some ERP components (e.g., P100, N200 and P300) were larger in the audiovisual condition than in the visual-only and auditory-only conditions for the target stimuli. The increased P100 was likely affected by audiovisual integration. Furthermore, in several time intervals, the difference between the target and nontarget responses was greater for the audiovisual stimuli than for the auditory-only and visual-only stimuli, as shown in Fig. 2. This enhanced difference was useful in improving the performance of the BCI (see Table 1).

Several previous studies support our observation that higher accuracy can be obtained in the audiovisual condition than that in the visual-only or auditory-only condition^15^,^29^,^43^. However, a previous study showed that an audiovisual BCI did not outperform a visual BCI^14^. There may be two possible reasons for this discrepancy. First, in the study, spoken stimuli were recognized over different time courses, which would affect the evocation of P300 responses. Second, the investigation was an offline study without feedback. Furthermore, each run lasted a long time (200 s), after which a short break (approximately 1 min) ensued. These factors may reduce subjects’ attention and affect their P300 responses.

Our paradigm was different from the classic “oddball” paradigm. Specifically, we adopted two stimuli because the recognition ability of patients with DOC is very low. The oddball effect was generated by randomly setting the time interval between two adjacent stimuli. Previous studies have demonstrated the effectiveness of such paradigms^44^,^45^. Our experimental results presented in Fig. 2 show that the target stimuli actually produced a P300 response. Although the nontarget stimuli also generated a P300, the response was much lower than that generated by the target stimuli. In the current study, the classification accuracy was much lower for the auditory-only condition than for the visual-only or audiovisual condition. One possible reason is that only two stimuli were used^14^.

As previously mentioned, misdiagnosis rates based on behavioral observation scales are relatively high. BCIs can be used to assess patients’ residual cognitive ability and, furthermore, as a supportive bedside tool. For instance, if awareness is detected in a VS patient using a BCI system, then the patient possesses the cognitive function associated with the experimental task and might be misdiagnosed. Future studies may also involve the development of a simple “yes/no” communication tool for patients with DOC based on the proposed BCI system.

Several auditory-only and visual-only BCIs for patients with DOC have been developed. Kubler *et al.* presented an auditory P300-based BCI speller for patients with LIS. Two of the four patients achieved a mean accuracy of 24%^46^, and the other two patients could not output any correct characters. Lule *et al.* reported that 1 MCS patient and 1 LIS patient among 18 patients with DOC could use a four-choice auditory P300-based BCI with accuracy higher than the chance level^38^. Pan *et al.* used a hybrid visual brain-computer interface combining P300 and SSVEP to detect awareness in patients with DOC^40^. Three (one VS, one MCS, and one LIS) among the eight DOC patients who were involved in the experiment achieved accuracies higher than the chance level. In this study, we applied a novel audiovisual BCI for awareness detection in patients with DOC. Our experimental results for healthy subjects show that the audiovisual BCI outperformed the corresponding visual-only and auditory-only BCIs, whereas the experimental results for the patients demonstrate the satisfactory performance of the system in awareness detection. Specifically, among the seven DOC patients (three VS and four MCS) who participated in the experiment, five (one VS and four MCS) obtained accuracies that were significantly higher than the chance level (Table 2). Furthermore, as shown in Fig. 4, the target stimuli elicited a P300 response, whereas the nontarget stimuli did not. To some extent, these results indicated both command following and residual number recognition ability in the five patients. Our BCI system may be suitable for DOC patients who have somewhat intact audition and vision. For a patient who lacks auditory abilities but has normal visual abilities, he/she can use our system as a visual BCI system. However, if a patient lacks visual abilities, our system will not work because the performance of the corresponding auditory BCI system is not sufficient for awareness detection.

In this study, we conducted experiments in several separate blocks because patients’ statuses did not allow them to perform many trials (e.g., more than 20) per session. Our findings suggest that patient status may have affected the classification results; that is, for some patients, their status varied between separate blocks, which caused variation in the classification results.

One could argue that false-positive results were obtained in this study. Indeed, false-positive findings in BCI studies are possible. With a statistical significance of *p* < 0.05, if 20 patients are tested independently, one of these patients may be falsely determined to be “aware”^47^,^48^. In this study, seven patients were tested and five of them were determined to be positive. Furthermore, in the binomial test, the maximum *p*-value of the five patients was approximately 0.01. Therefore, it is unlikely that false-positive results were generated. For patient P1 (VS), who showed significant accuracy, the behavioral assessment performed one month after the experiment indicated that his CRS-R score had significantly increased (from 5 during the experiment to 8). This finding supports our test results based on the BCI.

## Materials and Methods

### Subjects

Ten healthy subjects with normal/corrected-to-normal vision and normal audition and seven patients with DOC (six males; three VS and four MCS; mean ± SD, 37 ± 17 years; see Table 3) from a local hospital participated in the experiment. None of the patients had a history of impaired visual or auditory acuity. This study was approved by the Ethics Committee of Liuhuaqiao Hospital in Guangzhou, China, which complies with the Code of Ethics of the World Medical Association (Declaration of Helsinki). Written informed consent was obtained from the legal surrogate of each patient. The VS and MCS clinical diagnoses were based on the JFK CRS-R scale, which comprises six subscales addressing auditory, visual, motor, oromotor, communication and arousal functions^49^.

### Data acquisition

A NuAmps device (Compumedics, Neuroscan, Inc., Australia) was used to collect 30-channel scalp EEG signals with a sampling rate of 250 Hz. Each subject wore an EEG cap (LT 37), and the EEG signals were referenced to those obtained for the right ear. All electrode impedances were kept below 5 kΩ during data collection.

### Experimental procedure

The subjects were seated in a comfortable chair approximately 0.5 m from a 22-in LED monitor. The healthy subjects participated in Experiment I, and the patients participated in Experiment II.

Experiment I: Each healthy subject performed three experimental sessions, corresponding to the visual, auditory and audiovisual stimulus conditions, in a random order for counterbalance. In each session, the subject first performed a training run of 10 trials to collect training data. In this study, we collected a small training data set for each subject, since the BCI system was designed mainly for DOC patients who are easily fatigued during experiments. A support vector machine (SVM) model was obtained based on the training data, after which each subject performed a test run of 30 trials.

**Figure 5:**
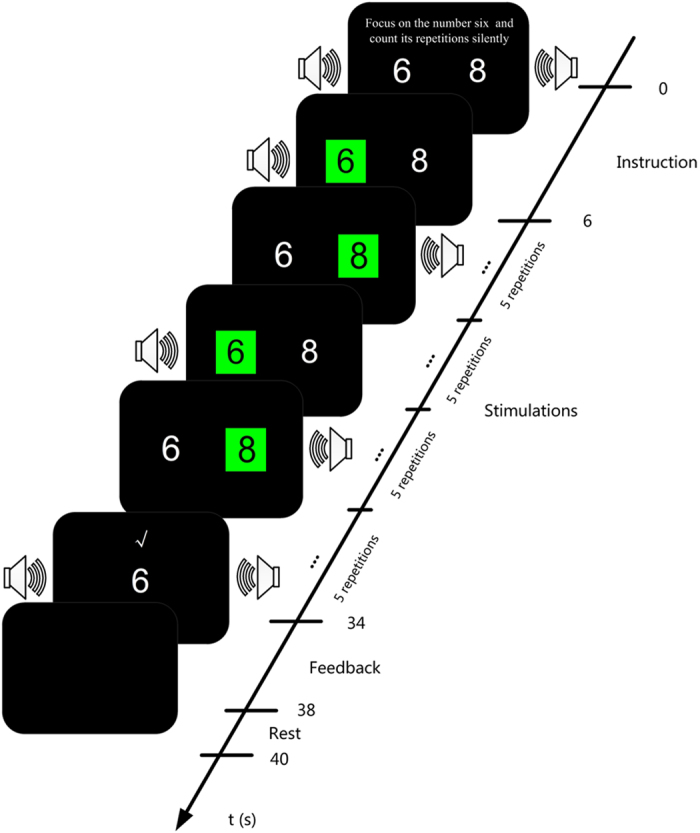
Procedure followed for one trial in the audiovisual session, including audiovisual instruction (0–6 s), audiovisual stimulations (6–34 s), feedback of classification result (34–38 s), and the rest period (e.g., 38–40 s). The audiovisual stimulations involved five repetitions of an audiovisual stimulus, e.g., target number 6; five repetitions of another audiovisual stimulus, e.g., nontarget number 8; five repetitions of the first audiovisual stimulus; and five repetitions of the second audiovisual stimulus. Note that each audiovisual stimulus lasted 300 ms, and the time interval between two adjacent audiovisual stimuli was randomly chosen from 700 ms, 900 ms, 1100 ms, 1300 ms, and 1500 ms.

The graphical user interface (GUI) and the experimental procedure of one trial for the audiovisual session are illustrated in Fig. 5. Two different numbers, randomly drawn from numbers 0–9 (e.g., 6 and 8 in Fig. 5), were presented on the left and right sides of the screen. Two speakers were placed on a table slightly lateral to and behind the monitor. Each trial began with audiovisual instruction in Chinese and lasted 6 s. The instructions were “Focus on the audiovisual target number (e.g., 6 in Fig. 5) and count its repetitions silently”. After the instruction, the two number buttons (size: 6.6 cm × 9 cm) alternately flashed on and off, during which the color of the flashing button changed from green to black. When a number button flashed, the corresponding spoken number was simultaneously presented from the ipsilateral speaker with a maximum sound-pressure level (SPL) of approximately 65 dB^41^. Each audiovisual stimulus lasted 300 ms. That is, the auditory and visual stimuli were temporally, spatially and semantically congruent. More specifically, there were two rounds of audiovisual stimulations following the instruction. In the first round, the audiovisual stimulus of one button (randomly chosen from the two buttons, e.g., 6 in Fig. 5) was presented five times, and then the audiovisual stimulus of the other button (e.g., 8 in Fig. 5) was repeated five times. The second round was the same as the first one. Note that the time interval between every two adjacent audiovisual stimuli was randomly chosen from 700, 900, 1100, 1300, and 1500 ms. If the target number was detected by the SVM model after stimulation, as feedback, the sound of applause was presented, and the detected number appeared for 4 s; otherwise, a cross appeared for 4 s. Finally, there was a 2 s break at the end of each trial.

For the visual session and auditory session, the experimental procedure was similar to that for the audiovisual session with one exception: there were visual-only stimuli for the visual session and auditory-only stimuli for the auditory session.

Experiment II: Seven patients participated in this experiment. Before the online experiment, each patient performed a calibration run of 10 trials. The test run contained five blocks, each of which was composed of 10 trials and was conducted on separate days because the patients were easily fatigued. For these patients, the test run lasted from one to two weeks. Using EEG data from the calibration run, we trained a SVM classifier for the first test block. The classification model was updated after each test block using the data from this block. For example, before Block 3, we used the data from Block 2 to re-train/update the SVM model for the test in Block 3. The procedure of each trial was similar to that for the audiovisual session in Experiment I. However, the experimenter and families explained the instructions repeatedly so that the patients paid attention to the audiovisual target stimuli. Additionally, the rest time between two adjacent trials was extended to at least 10 s depending on the patient’s level of fatigue. If the patient showed decreased arousal (i.e., eyes closed) in a trial, then this trial was discarded and the next trial began after the patient reawakened.

### Data processing

Classification algorithm: We performed the same online analysis for Experiments I and II as follows. For each trial of the training and testing runs, the EEG signals recorded from 30 channels were band-pass filtered (0.1–20 Hz). Then, for each channel, the data epoch corresponding to each stimulus was extracted from 0 to 500 ms after the stimulus onset. All epochs were referred to the mean amplitudes in the baselines that were computed from a −100 to 0 ms time interval before the stimulus onset. The epochs were downsampled at a rate of 5. Next, a data vector was obtained by concatenating the epochs from all 30 channels. Finally, a feature vector corresponding to each number was constructed by averaging the vectors from all repetitions. Note that the features used for classification encompassed some ERP components within 500 ms after stimulus onset, such as P100, N200 and P300. An SVM classifier was first trained using the training data, in which the feature vectors corresponding to the target and nontarget numbers were labeled + 1 and −1, respectively. For each test trial, the trained SVM was applied to the two feature vectors corresponding to the two numbers, and the predicted target number was that corresponding to the higher score.

Offline data analysis for Experiment I: We first performed ERP analyses using the 30 trials in the test run in each session. After band-pass filtering (0.1–55 Hz), the epoch data were extracted from −100 ms before to 500 ms after each stimulus onset. Baselines were computed from the −100 to 0 ms time interval prior to the stimulus onset. For artifact rejection, the epochs were discarded from averaging if the potential exceeded 100 μV in any one of channels. ERP responses were extracted by time-locked averaging the EEG signal across 30 trials in the test run for each stimulus type. To observe audiovisual integration, we compared the ERP response for the audiovisual session with the sum of the ERP responses for the visual-only and auditory-only sessions. We also compared the ERPs for the target and nontarget stimuli to illustrate the effectiveness of our audiovisual BCI system.

To determine the discriminative features in the three stimulus conditions, we performed point-wise running t-tests for target vs. nontarget responses in the audiovisual, visual-only, and auditory-only stimulus conditions across all healthy subjects, where a cluster size of 7 was used for multiple comparison correction^42^,^50^,^51^. We also used the features in three time windows containing P100, N200 and P300 for offline classification. For each time window and each stimulus condition, we performed a 10-fold cross-validation using the 30 trials of the test run to obtain the classification accuracies.

Performance measures for Experiment II: We used a binomial test based on Jeffreys’ Beta distribution to calculate the chance level in a two-class paradigm as follows^52^,^53^:


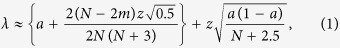


where *N* is the number of trials, *m* is the expected number of successful trials, *a* is the expected accuracy (0.5 in this study), λ is the accuracy rate, and *z* is the z-score based on the standard normal distribution. Given a significance level of 0.05 for a one-sided test, *z* is 1.65. Using (1), we could obtain the accuracy rate λ corresponding to the significance level, which was 62.7% and 61.4% for 40 and 50 trials, respectively.

## Additional Information

**How to cite this article**: Wang, F. *et al*. A Novel Audiovisual Brain-Computer Interface and Its Application in Awareness Detection. *Sci. Rep.* doi: 10.1038/srep09962 (2015).

## Figures and Tables

**Table 1:** Offline classification accuracies in different time windows for the audiovisual, visual-only and auditory-only modalities.

	**Classification accuracies (%)**											
**Subjects**	**100**–**200 ms**			**200**–**300 ms**			**300**–**500 ms**			**0**–**500 ms**		
	**AV**	**V**	**A**	**AV**	**V**	**A**	**AV**	**V**	**A**	**AV**	**V**	**A**
H1	70	56.67	73.33	100	96.67	33.33	96.67	93.33	56.67	96.67	100	43.33
H2	70	86.67	53.33	100	100	60	96.67	93.33	53.33	100	90	43.33
H3	93.33	96.67	33.33	93.33	100	53.33	73.33	83.33	56.67	90	96.67	56.67
H4	80	76.67	66.67	100	80	56.67	96.67	90	46.67	96.67	80	40
H5	100	96.67	56.67	100	90	80	76.67	70	63.33	100	90	56.67
H6	66.67	73.33	40	96.67	100	36.67	96.67	86.67	63.33	100	93.33	53.33
H7	93.33	83.33	30	100	96.67	40	100	86.67	76.67	100	93.33	70
H8	100	100	43.33	96.67	96.67	60	76.67	90	56.67	90	93.33	53.33
H9	90	83.33	50	100	90	66.67	100	86.67	46.67	100	93.33	80
H10	80	60	63.33	90	93.33	63.33	93.33	100	70	93.33	100	80
Mean	84.33	81.33	51	97.67	94.33	55	90.67	88	59	96.67	93	57.67

**Table 2:** Online accuracy for each patient.

**Patient**	**Trials**	**Hits**	**Accuracy**	***p-*****value**
P1	50	33	66%	0.0102
P2	50	26	54%	0.3860
P3	40	24	60%	0.0961
P4	50	37	74%	<0.001
P5	50	35	70%	0.0019
P6	40	27	67.5%	0.0113
P7	52	35	67.3%	0.0053

The significance levels for 40, 50 and 52 trials were determined to be 62.7%, 61.4%, and 61.2% in the binomial test, respectively (*p* < 0.05).

**Table 3:** Summary of patients’ clinical status.

**Patient**	**Age (years)**	**Gender**	**Clinical Diagnosis**	**Etiology**	**Time Since Onset (months)**	**JFK CRS-R score (subscores)**	
						**Before the experiment**	**After the experiment**
P1	43	M	VS	TBI	5	5 (1-0-2-1-0-1)	8 (1-1-2-2-0-2)
P2	28	F	VS	TBI	0.5	5 (1-0-1-1-0-2)	9 (1-3-2-1-0-2)
P3	51	M	VS	TBI	20	9 (2-1-2-2-0-2)	9 (2-1-2-2-0-2)
P4	48	M	MCS	TBI	3.5	15 (3-3-5-2-0-2)	16 (3-3-5-2-1-2)
P5	34	M	MCS	TBI	1.5	9 (1-1-5-1-0-1)	15 (3-4-5-1-0-2)
P6	37	M	MCS	TBI	4	9 (1-3-2-1-0-2)	9 (1-3-2-1-0-2)
P7	20	M	MCS	TBI	4	7 (1-0-3-1-0-2)	7 (1-0-3-1-0-2)

VS, vegetative state; MCS, minimally conscious state; and TBI, traumatic brain injury. JFK CRS-R subscales: auditory, visual, motor, oromotor, communication, and arousal functions.
